# Serum Neuropeptide Y and Ghrelin in Female Adolescents Exposed to Childhood Sexual Abuse with and Without Post-Traumatic Stress Disorder

**DOI:** 10.3390/children13101445

**Published:** 2026-10-21

**Authors:** Nihal Yurteri, Enes Sarıgedik, Rahime Kaya, İbrahim Ethem Şahin

**Affiliations:** 1 Department of Child and Adolescent Psychiatry, Faculty of Medicine, Kütahya Health Sciences University, Kütahya 43100, Türkiye; 2 Department of Child and Adolescent Psychiatry, Faculty of Medicine, Sakarya University, Sakarya 54290, Türkiye; 3 Department of Medical Biochemistry, Faculty of Medicine, Düzce University, Düzce 81620, Türkiye

**Keywords:** childhood sexual abuse, post-traumatic stress disorder, neuropeptide Y, ghrelin, adolescent

## Abstract

**Highlights:**

**What are the main findings?**

- Serum NPY concentrations were lower in female adolescents with CSA-related PTSD than in both CSA-exposed adolescents without PTSD and healthy controls.
- Serum ghrelin concentrations showed a graded pattern, with the highest levels in CSA-related PTSD, intermediate levels in CSA without PTSD, and the lowest levels in healthy controls.

**What are the implications of the main findings?**

- NPY and ghrelin may reflect distinct stress-related biological patterns among adolescents exposed to CSA, but the observed differences should not be considered specific to PTSD.
- Further longitudinal studies are needed to determine whether similar NPY and ghrelin patterns are observed in larger and more diverse samples.

**Abstract:**

Background: Childhood sexual abuse (CSA) is a major risk factor for post-traumatic stress disorder (PTSD), but biological factors associated with differing clinical outcomes following CSA remain poorly understood. We compared serum neuropeptide Y (NPY) and ghrelin concentrations in female adolescents with CSA-related PTSD, CSA without PTSD, and healthy controls. Methods: The study included 111 postmenarcheal female adolescents aged 12–18 years: 37 with CSA + PTSD, 37 with CSA without PTSD, and 37 healthy controls. PTSD and other psychiatric disorders were assessed using the K-SADS-PL-DSM-5-T. Fasting morning serum NPY and ghrelin concentrations were measured by ELISA. Group comparisons were performed, and exploratory ROC analyses assessed within-sample discrimination between CSA-exposed adolescents with and without PTSD. Results: Serum NPY differed significantly across groups (F(2,108) = 43.63, *p* < 0.001, η^2^ = 0.447), with lower concentrations in the CSA + PTSD group than in both comparison groups; the CSA without PTSD and healthy control groups did not differ significantly. Ghrelin also differed significantly across groups (H(2) = 85.95, *p* < 0.001, ε^2^ = 0.777), showing a graded pattern with the highest concentrations in the CSA + PTSD group, intermediate concentrations in the CSA without PTSD group, and the lowest concentrations in healthy controls. Exploratory ROC analyses yielded AUCs of 0.896 for NPY and 0.881 for ghrelin within the CSA-exposed sample. Conclusion: Serum NPY and ghrelin showed distinct cross-sectional group patterns in female adolescents with and without CSA-related PTSD. These findings should not be interpreted as PTSD-specific or clinically validated diagnostic effects. Longitudinal studies in independent, more representative samples are needed to clarify their temporal and clinical significance.

## 1. Introduction

Exposure to childhood sexual abuse (CSA) is linked to a greater likelihood of post-traumatic stress disorder (PTSD) during childhood and adolescence, with similar effect sizes reported for males and females [1]. CSA is also associated with other mental health difficulties, including depression, anxiety, suicidal ideation, and nonsuicidal self-injury [2]. Nevertheless, PTSD develops in only a proportion of individuals exposed to CSA, reflecting substantial variation in psychological outcomes after trauma [1,2]. Identifying reliable biomarkers of pediatric PTSD has remained difficult [3]. Cortisol and inflammatory markers have received considerable attention in this context, but results across studies have been inconsistent and their clinical utility remains unclear [3,4]. PTSD has been associated with alterations in several neurobiological systems, including the hypothalamic–pituitary–adrenal (HPA) axis and stress-responsive neuropeptide networks [5].

NPY is an important component of the neurobiological response to stress and is widely expressed in forebrain limbic and brainstem regions involved in the regulation of stress and emotional behaviors [6,7]. Experimental studies have linked NPY to stress responses, anxiety, fear, and autonomic regulation, and NPY has also been proposed as a potential factor contributing to resilience to stress [6,7]. Clinical studies have reported lower central and peripheral NPY concentrations in PTSD [8,9], although findings for peripheral NPY have not been uniform and chronic stress has also been associated with higher plasma NPY concentrations [8]. These findings suggest that NPY is involved in the biological response to stress, while changes in NPY may not be specific to PTSD.

Ghrelin is another peptide involved in the stress response that has been investigated in relation to trauma-related psychopathology. Beyond its established role in energy homeostasis, ghrelin has been implicated in HPA-axis function as well as neural processes related to fear, reward, and anxiety [10]. Population-based data from Wittekind et al. showed an association between CSA and elevated circulating ghrelin concentrations in adulthood, with the association being more pronounced in women [11]. However, another human study found lower fasting ghrelin concentrations following early-life stress, suggesting that ghrelin findings may vary across study populations, stress exposures, assay platforms, and clinical characteristics [12].

Biological research specifically addressing pediatric CSA remains relatively limited, particularly studies comparing adolescents exposed to CSA with and without PTSD [13]. Moreover, findings for cortisol, ACTH, BDNF, and oxidative stress measures have not shown a consistent separation between adolescents with PTSD and trauma-exposed adolescents who did not develop PTSD [13,14]. Based on previous evidence linking NPY and ghrelin to stress-related neurobiological processes, we hypothesized that serum concentrations of these neuropeptides would differ among adolescents with CSA-related PTSD, adolescents with CSA without PTSD, and healthy controls. Given the limited and heterogeneous evidence regarding the direction of these differences, particularly in adolescents exposed to CSA, the hypothesis was formulated as non-directional. The primary objective was therefore to compare serum NPY and ghrelin concentrations across these three groups. We additionally explored the ability of NPY and ghrelin to discriminate between CSA-exposed adolescents with and without PTSD using ROC analyses.

## 2. Materials and Methods

### 2.1. Participants

The study included postmenarcheal female adolescents aged 12–18 years. The study was restricted to female adolescents to reduce sex-related biological heterogeneity and to obtain a more homogeneous study sample for the comparison of serum NPY and ghrelin concentrations. Within this population, the sampling frame consisted of 139 female adolescents with a documented history of CSA who had undergone forensic evaluation at our department before initiation of the study. All 139 adolescents were recalled and assessed for study eligibility between 1 April and 28 June 2019. After application of the predefined inclusion and exclusion criteria, 65 adolescents were excluded and 74 remained eligible. All 74 eligible adolescents were included in the study, of whom 37 met DSM-5 diagnostic criteria for PTSD and 37 did not, as determined by the K-SADS-PL-DSM-5-T assessment.

CSA-exposed participants had previously been referred to our department by judicial authorities for forensic evaluation following alleged sexual abuse and had therefore been involved in the legal/forensic process related to the reported abuse. CSA exposure and abuse-related characteristics were ascertained from existing forensic and clinical records. The available abuse-related variables included single versus repeated abuse, presence of penetration, relationship to the perpetrator (intrafamilial, friend/romantic partner, or other), and the time elapsed between the most recent abuse episode and blood sampling. To minimize unnecessary repetition of potentially distressing experiences and the risk of retraumatization, participants were not asked to provide a detailed account of the sexual abuse again solely for research purposes.

**Figure 1:**
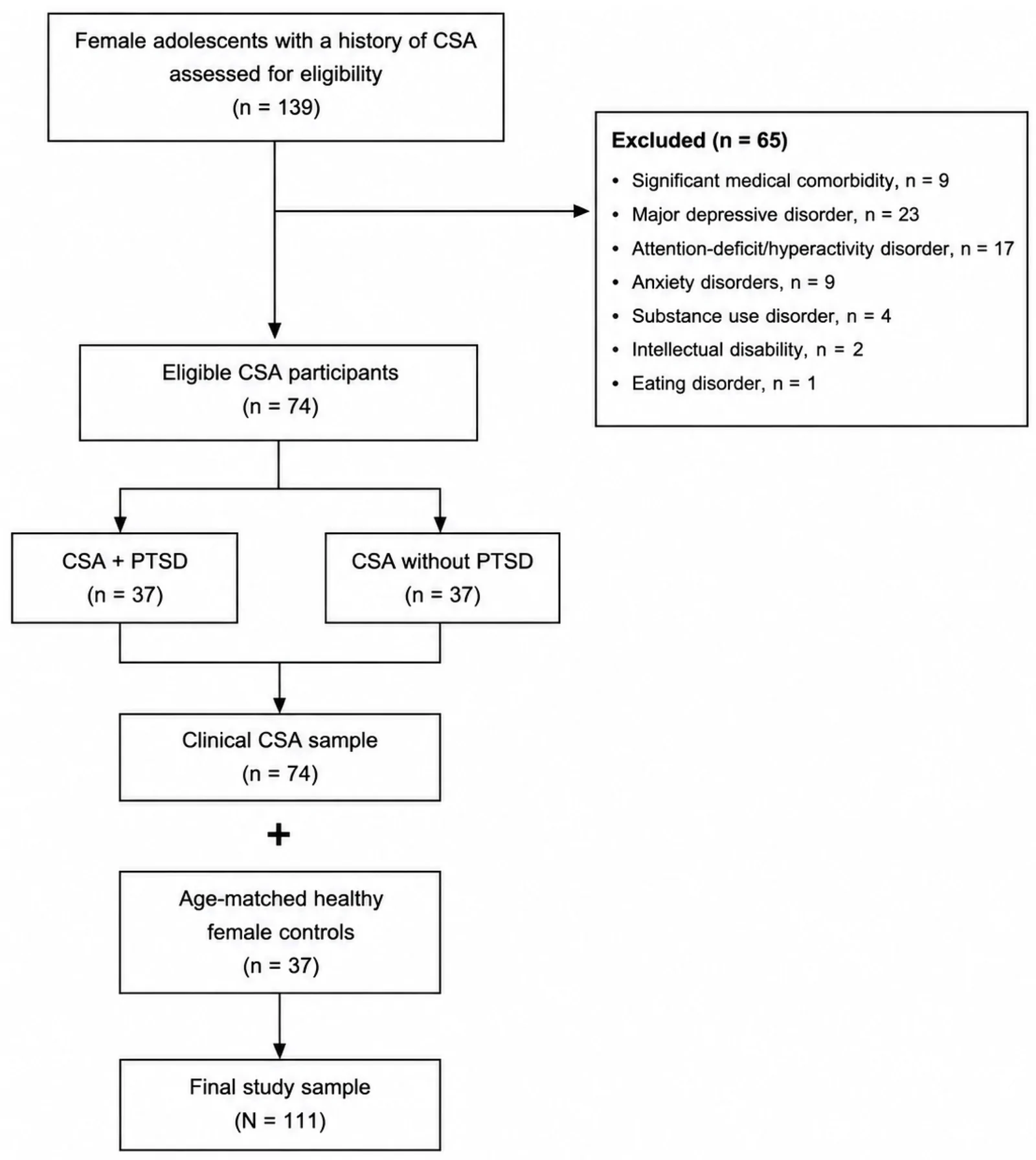
**Participant flow diagram.** To avoid double counting, participants meeting more than one exclusion criterion were classified according to the primary reason for exclusion.

Participants with significant medical comorbidities or comorbid psychiatric disorders, including major depressive disorder, intellectual disability, psychotic disorders, bipolar disorder, attention-deficit/hyperactivity disorder, autism spectrum disorder, obsessive–compulsive disorder, anxiety disorders, substance use disorders, and eating disorders, were excluded. The WISC-R was administered when intellectual disability was clinically suspected, and autism spectrum disorder was assessed clinically according to DSM-5 criteria. Reasons for exclusion are presented in Figure 1.

Participants in both CSA-exposed groups had no lifetime history of psychotropic medication use and had not used any medication during the 6 months preceding blood sampling, including hormonal contraceptives or other hormonal treatments. None had received formal psychotherapy; however, all CSA-exposed participants had received supportive clinical interviews following the abuse.

For the comparison group, healthy controls were selected from the control pool established for previous scale studies conducted at our center. Among adolescents in this pool, 37 participants were selected by individual age matching to the CSA + PTSD participants. All potential healthy controls were reassessed using the K-SADS-PL-DSM-5-T and the Turkish translation of the 29-item Traumatic Experiences Checklist (TEC) [15] (translated by Vedat Şar, 2002). Adolescents with any psychiatric disorder, significant medical conditions, or a history of traumatic exposure were excluded. Healthy controls had no lifetime history of psychotropic medication use, had not used any medication during the 6 months preceding blood sampling, and had never received psychotherapy or psychosocial support. Blood samples from the selected healthy controls were collected between 1 April and 28 June 2019, during the same period as those from the CSA-exposed participants. Serum NPY and ghrelin concentrations were measured in these samples for the first time specifically for the present study.

BMI-for-age z-scores (BMI-z) were calculated according to the World Health Organization (WHO) 2007 growth reference for girls aged 5–19 years. Regular education was defined as regular attendance in formal education at the grade level appropriate for the participant’s age. Low socioeconomic status was defined as a monthly family income below the national minimum wage applicable during the study period.

All participants underwent a structured psychiatric assessment using the K-SADS-PL-DSM-5-T, including the relevant trauma- and PTSD-related sections. Interviews were conducted by a trained child and adolescent psychiatrist who was also a study author, allowing immediate clinical assessment and intervention if psychological distress or other clinical concerns emerged. Written informed consent was obtained from the parent or legal guardian, together with assent from the adolescent. For participants for whom parental consent was not applicable, consent was obtained from the legally authorized representative responsible for the child. Participant data were de-identified and assigned coded study IDs by the same child and adolescent psychiatrist who conducted the diagnostic interviews. The dataset used for subsequent analyses contained no directly identifying information, and access to the coded research data was restricted to the study authors.

The study was conducted at the outpatient clinics of the Department of Child and Adolescent Psychiatry, Duzce University Faculty of Medicine. Ethics approval was obtained on 4 March 2019 (Medical Ethics Committee of Duzce University; approval no. 2019/45). All blood samples included in the present biomarker analyses were collected between 1 April and 28 June 2019, and the biochemical analyses were performed on 1 July 2019.

### 2.2. Measures

#### Schedule for Affective Disorders and Schizophrenia for School-Age Children–Present and Lifetime Version, DSM-5 (K-SADS-PL-DSM-5-T)

Psychiatric diagnoses were assessed using the Turkish version of the Schedule for Affective Disorders and Schizophrenia for School-Age Children–Present and Lifetime Version, DSM-5 (K-SADS-PL-DSM-5-T), a semi-structured diagnostic interview for current and lifetime psychiatric disorders in children and adolescents according to DSM-5 criteria [16,17]. The interview includes screening and disorder-specific supplementary sections, including those assessing trauma- and stressor-related disorders. In the present study, the K-SADS-PL-DSM-5-T was used to establish PTSD diagnosis and to assess psychiatric disorders specified in the eligibility criteria. A separate dimensional measure of PTSD symptom severity was not collected.

### 2.3. Blood Sampling and Analysis

Blood samples were collected from the antecubital vein between 08:30 and 10:30 after an overnight fast. Samples were allowed to clot at room temperature for 15 min and were then immediately centrifuged at 2500 rpm for 20 min at 4 °C. The separated serum was aliquoted and immediately stored at −80 °C until biochemical analysis. The maximum storage duration at −80 °C was approximately 3 months, and samples underwent one freeze–thaw cycle before analysis.

All serum samples were measured in duplicate. For each biomarker, samples from the three study groups were randomly distributed across three assay plates. Laboratory personnel performing the assays were blinded to clinical group and PTSD status, and samples were identified only by coded study IDs. For each biomarker, all three assay plates were from the same kit lot (lot no. 201901016). Assays were performed according to the manufacturer’s standard calibration procedures, and all measured concentrations were within the analytical ranges specified for the respective assays.

Serum NPY concentrations were measured using a commercially available human NPY ELISA kit (BT LAB, E1285Hu; Bioassay Technology Laboratory, Shanghai, China). The analytical range was 5–2000 pg/mL and the reported sensitivity was 2.36 pg/mL. According to the manufacturer, the intra-assay coefficient of variation was 3.7–5.7% and the inter-assay coefficient of variation was <10%.

Serum ghrelin concentrations were measured using a commercially available Human Ghrelin (GHRL) ELISA Kit (BT LAB, E3091Hu; Bioassay Technology Laboratory, Shanghai, China). The analytical range was 0.05–10 ng/mL and the reported sensitivity was 0.01 ng/mL. According to the manufacturer, the intra-assay coefficient of variation was <8% and the inter-assay coefficient of variation was <10%. Ghrelin concentrations were reported by the laboratory in ng/mL, and no subsequent unit conversion was performed for the statistical analyses or reporting of the results.

### 2.4. Statistical Analyses

Statistical analyses were performed using the Statistical Package for the Social Sciences (SPSS; IBM Corp., Armonk, NY, USA), version 20.0. The normality of continuous variables was assessed using the Shapiro–Wilk test together with visual inspection of histograms and Q–Q plots. Homogeneity of variances was assessed using Levene’s test. Continuous variables are presented as mean ± standard deviation (SD) or median (interquartile range [IQR]), as appropriate, and categorical variables as frequencies and percentages.

Continuous variables were compared across the three groups using one-way analysis of variance (ANOVA) or the Kruskal–Wallis test, as appropriate. Significant overall differences were followed by Tukey’s honestly significant difference (HSD) test or the Dunn–Bonferroni post hoc test, as appropriate. Categorical variables were compared using the chi-square test or Fisher’s exact test. Effect sizes were reported as eta squared (η^2^), epsilon squared (ε^2^), or Cramér’s V, as appropriate. BMI-z values were compared among the three groups using one-way ANOVA. Categorical CSA characteristics were compared between the CSA + PTSD and CSA without PTSD groups using Pearson’s chi-square test. The time elapsed between the most recent abuse episode and blood sampling was normally distributed and was compared using an independent-samples *t*-test. Biomarker distributions were additionally examined for observations outside the 1.5 × IQR boundaries. As a sensitivity analysis, the overall ghrelin group comparison was repeated after exclusion of the single observation above the upper 1.5 × IQR boundary.

Receiver operating characteristic (ROC) analyses were performed separately for serum NPY and ghrelin among the 74 CSA-exposed participants, with PTSD defined as the positive state. ROC analyses were considered exploratory. AUCs with 95% confidence intervals, data-derived cut-off values, sensitivity, specificity, and Youden index were used to characterize discrimination within the present sample and were not considered clinically validated diagnostic thresholds. All statistical tests were two-tailed, and a *p* value < 0.05 was considered statistically significant.

Given the fixed final sample size, a sensitivity power analysis was performed using G*Power 3.1. For a one-way ANOVA with three groups, a total sample of 111 participants, α = 0.05, and 80% power corresponded to a minimum detectable effect size of approximately Cohen’s f = 0.30.

## 3. Results

The participant flow and reasons for exclusion are shown in Figure 1. BMI-z did not differ significantly among the three study groups (one-way ANOVA, *p* = 0.899). Available CSA-related characteristics are presented in Table 1. No statistically significant between-group differences were observed in repeated abuse, penetration, perpetrator relationship, or the time elapsed between the most recent abuse episode and blood sampling (all *p* > 0.05).

Serum NPY concentrations differed significantly across the three groups, with lower concentrations in the CSA + PTSD group than in both the CSA without PTSD and healthy control groups. NPY concentrations did not differ significantly between the CSA without PTSD and healthy control groups (Table 2 and Table 3).

**Figure 2:**
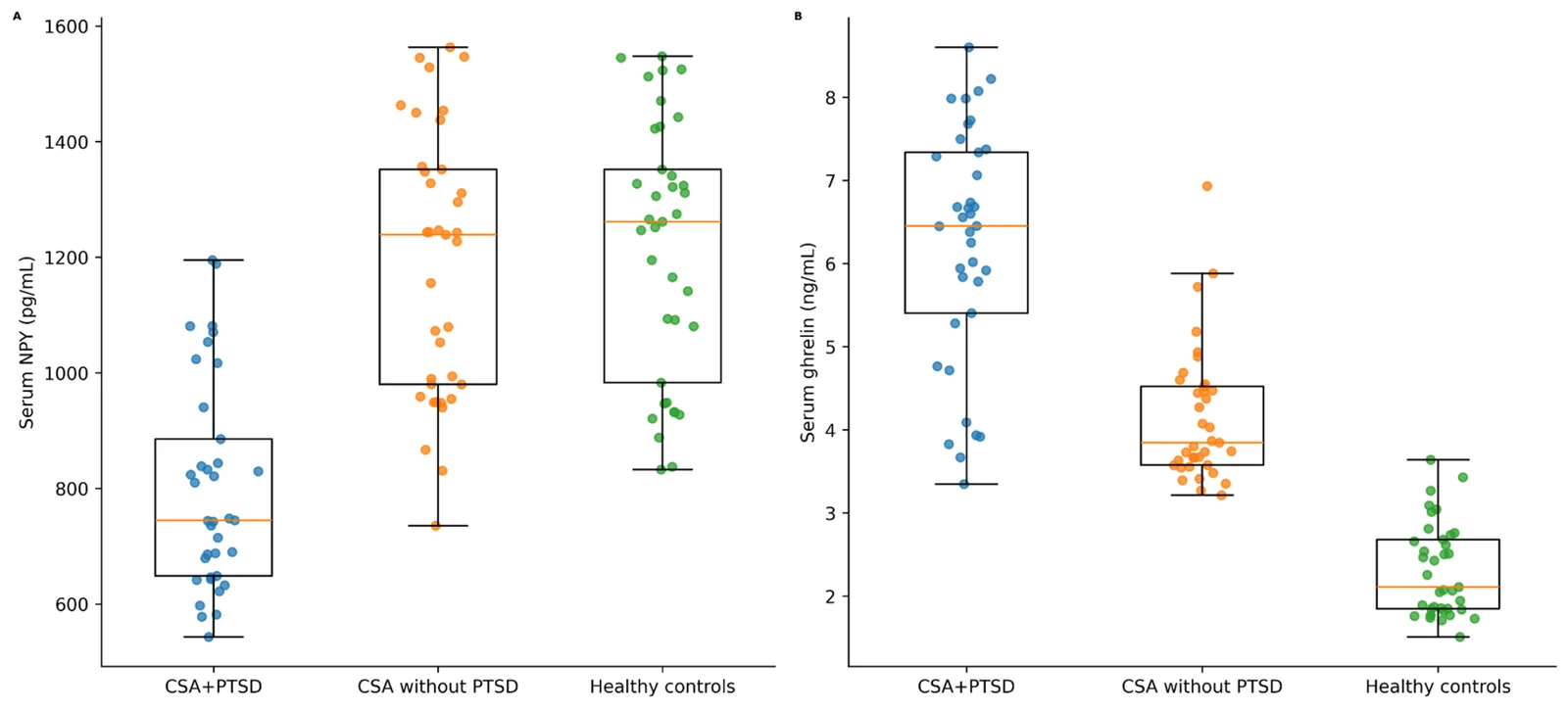
Individual distributions of serum NPY and ghrelin concentrations across the study groups. (**A**) Serum neuropeptide Y (NPY) concentrations and (**B**) serum ghrelin concentrations in adolescents with childhood sexual abuse (CSA) and PTSD, adolescents with CSA without PTSD, and healthy controls. Boxes indicate the median and interquartile range, whiskers extend to 1.5 times the interquartile range, and individual points represent individual participants (n = 37 per group).

Serum ghrelin concentrations also differed significantly across groups, showing a graded pattern with the highest concentrations in the CSA + PTSD group, intermediate concentrations in the CSA without PTSD group, and the lowest concentrations in healthy controls (Table 2 and Table 3). No observations exceeded the 1.5 × IQR boundaries for NPY. For ghrelin, one observation (6.93 ng/mL) in the CSA without PTSD group exceeded the upper 1.5 × IQR boundary. A sensitivity analysis excluding this observation yielded an essentially unchanged overall group difference (H(2) = 86.44, *p* < 0.001). The individual distributions of serum NPY and ghrelin concentrations across the three groups are shown in Figure 2.

**Figure 3:**
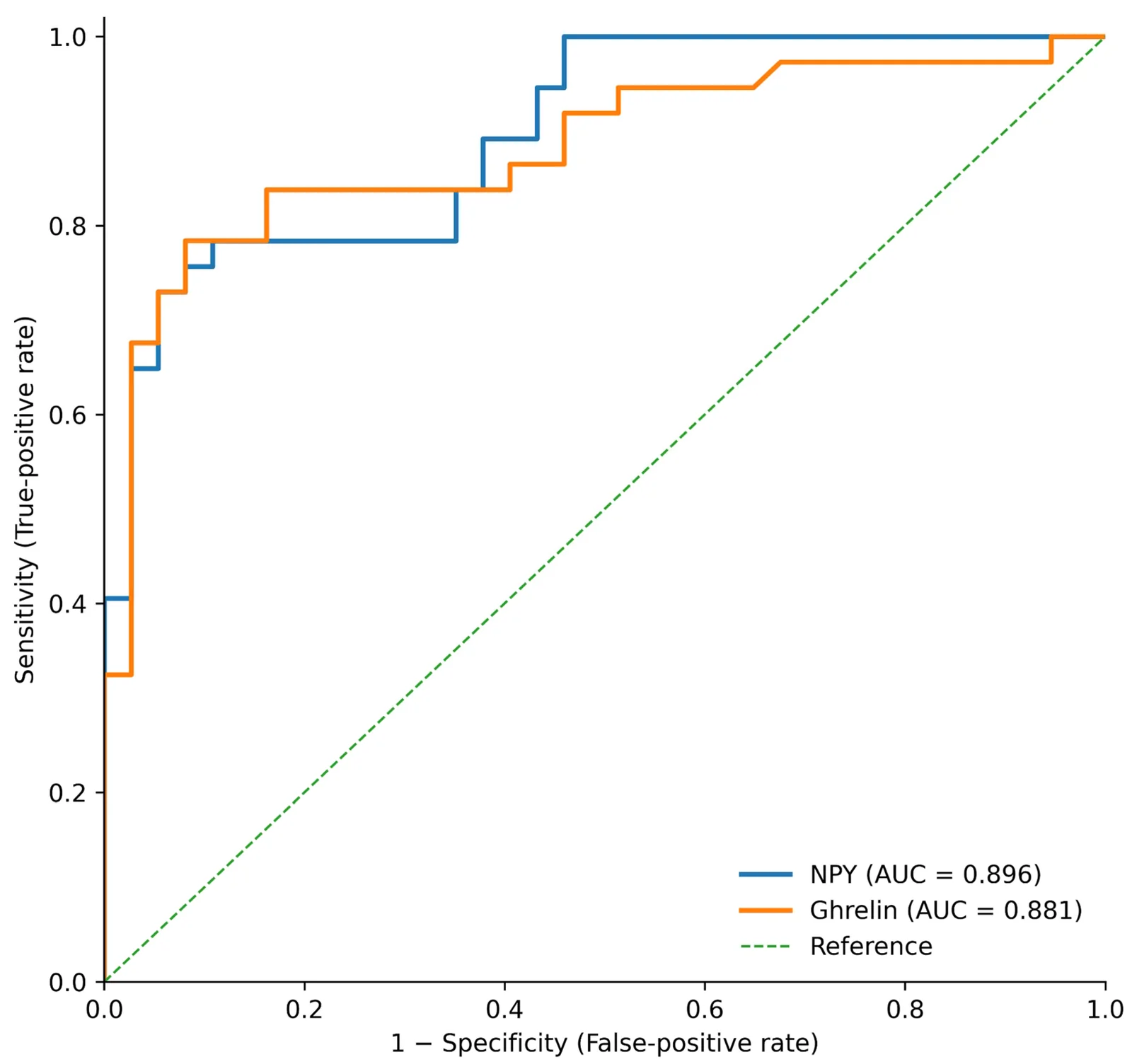
Receiver operating characteristic (ROC) curves for serum NPY and ghrelin in distinguishing PTSD among adolescents exposed to childhood sexual abuse (CSA). ROC analyses included the 74 CSA-exposed participants (CSA + PTSD, n = 37; CSA without PTSD, n = 37), with PTSD defined as the positive state. Serum neuropeptide Y (NPY) yielded an area under the curve (AUC) of 0.896, and serum ghrelin yielded an AUC of 0.881. The ROC analyses were exploratory, and the resulting AUCs and data-derived cut-off values represent discrimination within the present sample rather than clinically validated diagnostic thresholds.

In exploratory ROC analyses restricted to the CSA-exposed participants, both NPY and ghrelin showed discrimination between participants with and without PTSD. The data-derived cut-off values are reported as exploratory thresholds within the present sample (Figure 3 and Table 4).

## 4. Discussion

In this study, serum NPY and ghrelin showed distinct cross-sectional patterns across female adolescents with CSA + PTSD, adolescents with CSA without PTSD, and healthy controls. NPY concentrations were lower in the CSA + PTSD group than in both comparison groups, whereas the CSA without PTSD and healthy control groups did not differ significantly. Ghrelin showed a graded pattern across the three groups. These findings should be interpreted as group associations rather than as evidence of PTSD-specific biological effects.

The lower NPY concentrations observed in the CSA + PTSD group are broadly consistent with previous reports of reduced NPY in PTSD [8,9,18,19]. NPY is implicated in neural systems involved in fear, anxiety, autonomic regulation, and stress adaptation [6,7,20], but peripheral NPY findings have not been uniform. In contrast, Nishi et al. did not identify a relationship between serum NPY concentrations and PTSD at one month following serious motor vehicle accidents [21]. Differences in trauma type, timing, biological compartment, and study population may contribute to this heterogeneity.

The similar NPY concentrations observed in the CSA without PTSD and healthy control groups are hypothesis-generating and should not be interpreted as evidence of a protective or resilience-related mechanism. Although this pattern may be consistent with previous reports suggesting a possible relationship between NPY and stress resilience or recovery [22,23,24], alternative explanations include individual differences in biological responses to trauma, unmeasured trauma characteristics, and other unmeasured biological or developmental factors. Prospective evidence from two large military cohorts showed that neither pre-deployment plasma NPY concentrations nor longitudinal plasma NPY trajectories were associated with subsequent PTSD symptom trajectories [25]. Thus, peripheral NPY has not been established as a prospective marker of resilience or subsequent PTSD development. Because the present study was cross-sectional, it cannot determine whether the observed NPY pattern preceded trauma exposure or PTSD development, changed after trauma, or reflected other individual characteristics.

The ghrelin findings also require cautious interpretation. The graded pattern of ghrelin concentrations across the three groups should not be interpreted as specific to PTSD. Ghrelin concentrations may reflect responses to trauma exposure as well as PTSD status. Previous studies have reported heterogeneous associations between stress exposure and circulating ghrelin, including higher ghrelin in adults with a history of CSA [11], higher acyl-ghrelin in adolescents with severe trauma and PTSD [26], and lower fasting ghrelin in young adults with early-life stress [12]. Differences in developmental stage, trauma exposure, clinical characteristics, and assay methodology may contribute to these contrasting findings.

The two CSA-exposed groups did not differ significantly in the abuse-related characteristics available in the present study, including repeated abuse, penetration, perpetrator relationship, and time elapsed since the most recent abuse episode. However, the absence of statistically significant differences in these measured characteristics does not establish equivalence of overall trauma exposure. CSA is a heterogeneous experience, and other dimensions of trauma severity and chronicity were not systematically available for analysis. Therefore, residual confounding by unmeasured trauma characteristics cannot be excluded, and the observed differences in NPY and ghrelin cannot be attributed specifically to PTSD independently of all characteristics of the traumatic exposure.

Direct comparison of ghrelin concentrations across studies should also be made cautiously because measured concentrations may vary according to assay platform and the molecular form of ghrelin assessed. In the present study, the available assay documentation did not establish that the assay measured total ghrelin or a specific molecular form, including acyl-ghrelin. Therefore, the findings are reported as serum ghrelin and are not interpreted as representing a specific molecular form.

Prospective and longitudinal studies provide additional context for interpreting these cross-sectional findings. As noted above, prospective military-cohort data did not support peripheral NPY as a simple predictor of subsequent PTSD symptom trajectories [25]. In contrast, longitudinal work has reported persistent elevations of circulating acyl-ghrelin years after severe adolescent stress exposure [27]. Because that study assessed acyl-ghrelin, it should not be considered a direct replication of the present assay. Together, these findings illustrate that stress-related peptide alterations may precede, accompany, or persist after stress exposure, whereas the single-time-point design of the present study cannot distinguish among these possibilities.

The ROC findings should be interpreted as exploratory measures of within-sample discrimination rather than evidence of diagnostic validity. Because the cut-off values were derived and evaluated in the same relatively small sample, their performance may be affected by optimism bias. Accordingly, these thresholds should not be considered clinically validated and require evaluation in an independent cohort before any potential diagnostic application can be considered. Moreover, the balanced distribution of PTSD and non-PTSD participants in the present sample does not reflect the prevalence of PTSD in broader CSA-exposed populations, further limiting the clinical interpretation of classification performance.

The exclusion of participants with psychiatric comorbidities was intended to reduce potential confounding by co-occurring psychiatric disorders but resulted in a clinically selected sample. Since psychiatric comorbidities may occur in adolescents with CSA-related PTSD, the study sample may not fully represent the broader clinical population. The restrictive eligibility criteria may also have narrowed the clinical spectrum represented in the study, and the findings should therefore be generalized beyond this selected population with caution.

Several limitations should be considered when interpreting the present findings. First, although available CSA-related characteristics, including repeated abuse, penetration, perpetrator relationship, and time elapsed since the most recent abuse episode, were examined, these variables did not capture all dimensions of trauma exposure. The absence of statistically significant differences in the measured characteristics does not establish equivalence of overall trauma exposure, and residual confounding by unmeasured trauma characteristics cannot be excluded. Second, PTSD was assessed categorically, and a separate dimensional measure of PTSD symptom severity was not collected; therefore, relationships between biomarker concentrations and PTSD symptom burden could not be examined. Third, although all participants were postmenarcheal, BMI-z was assessed, and none had used hormonal contraceptives or other hormonal treatments during the 6 months preceding blood sampling, Tanner stage, age at menarche, and menstrual-cycle phase were not systematically recorded and therefore could not be evaluated as potential sources of biomarker variability. Because the study included only female adolescents, the findings cannot be generalized directly to male adolescents. Fourth, the exclusion of psychiatric comorbidities resulted in a clinically selected, comorbidity-free sample that may not fully represent the broader population of adolescents with CSA-related PTSD, thereby limiting generalizability and potentially introducing spectrum bias. Healthy controls were screened for traumatic exposure using the Traumatic Experiences Checklist; however, the assessment of trauma history differed from the forensic and clinical documentation available for the CSA-exposed groups. Fifth, the original study-specific assay variability records are no longer available; therefore, study-specific intra-assay and inter-plate coefficients of variation cannot be reliably reported retrospectively. Sixth, no multivariable adjustment was performed. Given the relatively small sample size and the number of potentially relevant clinical and biological covariates, we retained the primary group comparisons rather than adding a post hoc multivariable model because of the risk of overfitting and unstable adjusted estimates. Consequently, residual confounding cannot be excluded. Seventh, the relatively small sample size and single-center design may limit the precision and generalizability of the findings. Finally, the ROC analyses were exploratory, and the data-derived cut-off values were both derived and evaluated in the same relatively small, balanced sample of CSA-exposed participants. Their apparent performance may therefore be affected by optimism bias and should not be interpreted as evidence of clinical diagnostic validity. Independent validation in larger and more representative cohorts is required before any potential clinical application can be considered. In addition, the cross-sectional design precludes conclusions regarding the temporal sequence or causal relationships among trauma exposure, PTSD, and the observed biomarker concentrations.

Our findings should also be considered in the context of the limited pediatric literature on biomarkers following CSA. Earlier pediatric CSA studies have evaluated cortisol, ACTH, BDNF, oxidative stress, and related biological measures, but these markers have not consistently differentiated participants with PTSD from trauma-exposed participants without PTSD [13,14]. The NPY and ghrelin differences observed in the present study extend this limited pediatric literature but do not establish diagnostic specificity. These findings support further investigation of NPY and ghrelin in adolescents exposed to CSA.

Including both adolescents with CSA without PTSD and healthy controls allowed us to examine the findings in relation to both trauma exposure and PTSD. The all-female sample, exclusion of major psychiatric and medical comorbidities, and standardized fasting morning blood collection also reduced potential sources of heterogeneity.

## 5. Conclusions

In conclusion, serum NPY and ghrelin concentrations differed across the study groups, with lower NPY and higher ghrelin in female adolescents with CSA + PTSD than in adolescents with CSA without PTSD. The CSA without PTSD group showed NPY concentrations similar to healthy controls and intermediate ghrelin concentrations. These cross-sectional findings do not establish a protective NPY mechanism, a PTSD-specific biomarker effect, or clinical diagnostic validity. Longitudinal studies in larger, more representative and independently validated samples are needed to clarify the temporal and clinical significance of these observations.

## Figures and Tables

**Table 1:** Demographic and CSA-related characteristics of the study groups.

Characteristic	CSA + PTSD (n = 37)	CSA Without PTSD (n = 37)	Healthy Controls (n = 37)	*p* Value
Age, years, median (IQR)	16.0 (14.0–17.0)	15.0 (14.0–16.5)	15.5 (14.5–17.0)	0.521
BMI-for-age z-score (WHO), mean ± SD	0.13 ± 0.69	0.10 ± 0.54	0.06 ± 0.72	0.899
Regular education, n (%)	29 (78.4)	30 (81.1)	32 (86.5)	0.653
Parents living together, n (%)	28 (75.7)	31 (83.8)	32 (86.5)	0.453
Low socioeconomic status, n (%)	11 (29.7)	8 (21.6)	6 (16.2)	0.375
**CSA characteristics**				
Repeated abuse, n (%)	14 (37.8)	11 (29.7)	—	0.461
Penetration, n (%)	12 (32.4)	10 (27.0)	—	0.611
Perpetrator relationship, n (%)				0.649
Intrafamilial	6 (16.2)	5 (13.5)	—	
Friend/romantic partner	26 (70.3)	24 (64.9)	—	
Other	5 (13.5)	8 (21.6)	—	
Time from most recent abuse to blood sampling, months, mean ± SD	11.0 ± 3.2	12.0 ± 4.1	—	0.246

Data are presented as median (interquartile range [IQR]), mean ± SD, or n (%), as appropriate. Age was compared using the Kruskal–Wallis test; BMI-for-age z-scores were compared using one-way ANOVA. Categorical demographic and CSA variables were compared using Pearson’s chi-square test. Time from the most recent abuse episode to blood sampling was compared using an independent-samples *t*-test. CSA characteristics were compared only between the two CSA-exposed groups. All tests were two-tailed. CSA, childhood sexual abuse; PTSD, post-traumatic stress disorder; BMI, body mass index; WHO, World Health Organization.

**Table 2:** Serum NPY and ghrelin concentrations across study groups.

Variable	CSA + PTSD (n = 37)	CSA Without PTSD (n = 37)	Healthy Controls (n = 37)	Test Statistic	*p* Value	Effect Size
NPY (pg/mL), mean ± SD	801.11 ± 180.17	1185.43 ± 230.81	1213.93 ± 222.45	F(2,108) = 43.63	<0.001	η^2^ = 0.447
Ghrelin (ng/mL), median (IQR)	6.45 (5.40–7.34)	3.85 (3.58–4.52)	2.11 (1.85–2.68)	H(2) = 85.95	<0.001	ε^2^ = 0.777

NPY was compared using one-way ANOVA; ghrelin was compared using the Kruskal–Wallis test. IQR, interquartile range; η^2^, eta squared; ε^2^, epsilon squared.

**Table 3:** Post hoc pairwise comparisons.

Biomarker	Pairwise Comparison	Estimate	95% CI	Adjusted *p* Value
NPY	CSA + PTSD vs. CSA without PTSD	−384.32 pg/mL	−501.62 to −267.02	<0.001
NPY	CSA + PTSD vs. healthy controls	−412.82 pg/mL	−530.12 to −295.52	<0.001
NPY	CSA without PTSD vs. healthy controls	−28.50 pg/mL	−145.80 to 88.80	0.833
Ghrelin	CSA + PTSD vs. CSA without PTSD	Dunn z = 3.825	—	<0.001
Ghrelin	CSA + PTSD vs. healthy controls	Dunn z = 9.226	—	<0.001
Ghrelin	CSA without PTSD vs. healthy controls	Dunn z = 5.401	—	<0.001

NPY pairwise comparisons were performed using Tukey HSD; ghrelin comparisons were performed using Dunn tests with Bonferroni adjustment. NPY, neuropeptide Y; PTSD, post-traumatic stress disorder; CI, confidence interval.

**Table 4:** Exploratory ROC analysis of serum NPY and ghrelin among CSA-exposed participants.

Biomarker	AUC	95% CI	Data-Derived Cut-Off	Sensitivity	Specificity	Youden J
NPY	0.896	0.827–0.965	≤855.42 pg/mL	73.0%	94.6%	0.676
Ghrelin	0.881	0.800–0.963	≥5.23 ng/mL	78.4%	91.9%	0.703

ROC analyses included the 74 CSA-exposed participants (CSA + PTSD, n = 37; CSA without PTSD, n = 37), with PTSD defined as the positive state. Cut-off values were derived from the present sample using the Youden index. These analyses were exploratory; the AUCs and cut-off values represent within-sample discrimination and should not be interpreted as clinically validated diagnostic thresholds. ROC curves are shown in Figure 3. AUC, area under the curve; CI, confidence interval; CSA, childhood sexual abuse; NPY, neuropeptide Y; PTSD, post-traumatic stress disorder.

## Data Availability

The participant-level data are not publicly available because they contain highly sensitive information concerning sexual abuse in minors, including forensic and psychiatric data. Due to the scope of the informed consent and ethical approval under which the data were collected, the dataset cannot be shared with external researchers.
